# Clinical Analysis of Cervical Lymph Node Metastasis Risk Factors and the Feasibility of Prophylactic Central Lymph Node Dissection in Papillary Thyroid Carcinoma

**DOI:** 10.1155/2021/6635686

**Published:** 2021-01-31

**Authors:** Yifan Chen, Shuo Chen, Xiaoying Lin, Xiangqing Huang, Xiaofang Yu, Juying Chen

**Affiliations:** ^1^Department of General Surgery, South Branch of Fujian Provincial Hospital, Fuzhou 350000, Fujian, China; ^2^Provincial Clinical Medical College of Fujian Medical University, Fuzhou 350000, Fujian, China; ^3^Department of Gynaecology and Obstetrics, South Branch of Fujian Provincial Hospital, Fuzhou 350000, Fujian, China; ^4^Department of Anesthesiology, South Branch of Fujian Provincial Hospital, Fuzhou 350000, Fujian, China

## Abstract

**Objective:**

To identify the risk factors for cervical lymph node metastasis (CLNM) and the feasibility of prophylactic central lymph node dissection.

**Methods:**

The characteristics of 1107 patients were extracted and analyzed. Univariate and multivariate analyses were used to identify risk factors associated with lymph node metastasis. The relationship between the central lymph node dissection (CLND) and lateral lymph node metastasis (LLNM) was analyzed using the correlation analysis.

**Results:**

The probability of CLNM was closely related to the male gender, age <55, and the increase of tumor size. Those patients with an increase in tumor size and CLNM were extremely prone to LLNM. Also, LLNM was more likely to happen in those with the more positive central lymph nodes. Routine prophylactic central lymph node dissection (P-CLND) did not increase the risk of complications.

**Conclusion:**

P-CLND should be considered as a reasonable surgical treatment for PTC.

## 1. Introduction

Following the improvements of ultrasonography (US) and US-guided fine-needle aspiration biopsy (FNAB), the incidence of thyroid carcinoma has been increasing over recent decades. Papillary thyroid carcinoma (PTC) is the most common thyroid malignancy. As an indolent disease, the majority of patients with PTC have an excellent 10-year prognosis with a survival rate of more than 99% [1]. The favorable prognosis results in controversy regarding the optimal therapeutic strategy for PTC. Some researchers, such as Ito, propose close follow-up for PTC patients instead of surgery [2]. Sugitani believes that delayed surgery has no effect on the outcome [3]. However, according to other studies, lymph node metastasis (LNM), especially cervical lymph node metastasis (CLNM), occurs in over 50% of patients with PTC [4, 5]. It is possible that besides being an independent risk factor for lateral lymph node metastasis (LLNM) [6–9], CLNM also elevates the recurrence rate and disease-specific mortality [10–12]. Repeated surgery may lead to an increase of complications, such as damage to the recurrent laryngeal nerve and hypoparathyroidism, which affect the life quality of patients. Central lymph node dissection (CLND) is recommended for patients who are suspected of CLNM in preoperative assessment. Yet, prophylactic central lymph node dissection (P-CLND) for patients with clinically node-negative is still controversial.

In the present study, we performed a retrospective analysis that focused on PTC patients without clinical evidence for CLNM, who received P-CLND. The aim of the study was to analyze the incidence, pattern, and risk factors of lymph node metastasis in PTC patients, to identify the high-risk patients and evaluate postoperative complications of P-CLND, so as to provide new evidence for the treatment of PTC patients.

## 2. Materials and Methods

### 2.1. Patients

A total of 1107 PTC patients who underwent thyroidectomy and P-CLND with or without lateral lymph node dissection between January 2015 and December 2019 at the Department of General Surgery, Fujian Provincial Hospital, were included in the study. Patients were enrolled according to the following criteria: (1) all cases that were preoperatively suspected as PTC following US examination, part of which were confirmed by FNAB—US characteristics include ultrasound intensity, composition, echoic distribution, tumor border, shape, calcification, aspect ratio, and blood flow; neck/thorax computer tomography (CT) is not systematically performed in our hospital—(2) all patients without clinical evidence of CLNM, who underwent unilateral or bilateral thyroidectomy with P-CLND and with or without lateral lymph node dissection; (3) all patients who were proven to have PTC by intraoperative frozen and postoperative pathological examination (all specimens were independently examined by two pathologists); and (4) all patients who were without a history of previous thyroid or neck surgery.

The study was approved by the Institutional Review Board of Fujian Provincial Hospital.

### 2.2. Treatment

In our hospital, P-CLND is routinely performed for PTC patients. Ipsilateral lobe and isthmus resection was performed for unilateral primary lesions. Total thyroidectomy was performed for bilateral primary lesions, and unilateral primary lesions requiring iodine-131 treatment. Lateral lymph node dissection is performed only if there is radiographic, cytopathologic, or intraoperative frozen pathological evidence suggestive of lymph node metastasis. Central lymph nodes include pretracheal, paratracheal, and prelaryngeal lymph nodes and lymph nodes located along the recurrent laryngeal nerve. Lateral lymph nodes include levels I, II, III, IV, and V. Levels II, III, and IV are routinely dissected. Levels I and V are dissected when lymph nodes metastases are suspected or confirmed. All patients received thyroid-stimulating hormone (TSH) suppression with levothyroxine (L-T4) after surgery. Part of the patients underwent iodine-131 treatment.

#### 2.2.1. Assessment of Surgical Complications

Serum parathyroid hormone (PTH) and total serum calcium were measured on the first postoperative day and every week for 3 consecutive weeks thereafter. Hypoparathyroidism (hypoPTH) was considered permanent when total serum calcium and PTH were still under normal value, or symptoms still existed at 6 months after surgery. Direct fiber-optic laryngoscopy was performed in all patients before and after surgery, to assess cord motility. Recurrent laryngeal nerve injury was considered permanent when the cord palsy persisted 6 months after surgery.

#### 2.2.2. Statistical Analysis

All statistical analyses were performed using the Statistical Package for Social Sciences (SPSS, Inc., Chicago, IL, USA). Univariate and multivariate analyses were performed to determine the significance of clinical characteristics. The univariate analyses were performed using the Chi-square test and Fisher's exact test. Variables with *P* < 0.05 in the univariate analysis were included in the multivariate analysis. The multivariate analyses were performed using binary logistic regression analysis. The results are presented as odds ratios (OR) with 95% confidence intervals (CI) and *P* values. A *P* value of <0.05 was considered statistically significant.

## 3. Results

There were 1107 patients enrolled in this study, including 828 females (74.8%) and 279 males (25.2%). In most of them, PTC was incidentally found during regular medical examinations. Others had symptoms, such as anterior neck mass, voice change, and lateral neck mass. The age of patients at the diagnosis time ranged from 15 to 80 years. Diameter ranged from 0.1 to 5.0 cm. 384 patients had a tumor size ≤5 mm (34.7%), 366 patients had a tumor size >5 mm but ≤10 mm (33.1%), 294 patients had a tumor size >10 mm but ≤20 mm (26.6%), and 63 patients had a tumor size >20 mm (5.6%). Among all patients, 225 patients exhibited multifocality in one thyroid lobe, and 171 patients had bilaterality. A total of 120 patients had adjacent structure invasion, such as strap muscles, recurrent laryngeal nerve. A total of 195 patients had Hashimoto thyroiditis.

After postoperative pathological examinations, LNM was present in 474 patients. Among them, 453 patients had CLNM (95.6%), 117 patients had LLNM (24.7%), and 21 patients had lateral without central lymph node metastasis. In this study, all patients received P-CLND. Seven hundred eighty-nine patients underwent lobectomy plus ipsilateral CLND, 174 patients underwent total thyroidectomy plus bilateral CLND, and 144 patients underwent total thyroidectomy plus bilateral CLND and unilateral LLND. The patients' characteristics are summarized in Table 1.

Table 2 shows the postoperative complications. Seventy-six patients had hypoparathyroidism that resolved within 3–6 months in 67 patients (6%) and was persistent in 9 patients (2.8%). Transient hypoparathyroidism was observed in 60 patients undergoing total thyroidectomy and in seven patients having lobectomy. Persistent hypoparathyroidism was observed in all patients who underwent total thyroidectomy. Among 34 cases with vocal cord palsy, which implied recurrent laryngeal nerve injury, 22 (2%) recovered within 1 to 6 months while the other 12 (1.1%) patients had a persistent injury. Two patients (0.2%) experienced bleeding problems after surgery, which led to reoperation. Chyle leakage, which was cured within 20 days, was observed in 5 patients (3.5%) undergoing total thyroidectomy plus CLND and LLND. None of the patients in this study had hematoma, tracheal leak, Horner's syndrome, and other complications.

**Figure 1:**
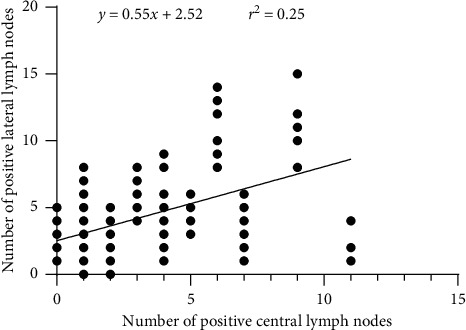
There is a positive correlation between the number of positive central and lateral lymph nodes.

**Figure 2:**
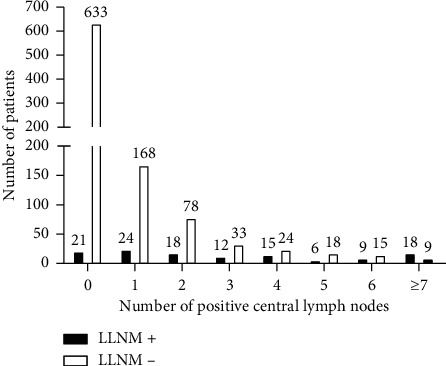
The incidence of lateral lymph node metastasis increased with the number of positive central lymph nodes. LLNM, lateral lymph node metastasis.

We first analyzed the relationship between clinical characteristics and LNM. Univariate analysis revealed that male gender, age <55, the increase in tumor size, multifocality, bilaterality, and extrathyroidal extension were significant predictors, but not Hashimoto thyroiditis. Then, multivariate logistic regression analysis was used to confirm the independently predictive factors of LNM. As shown in Table 3, the male gender, age <55, the increase in tumor size, and multifocality still resulted as independent factors. In the univariate analysis, CLNM was significantly associated with male gender, age <55, the increase in tumor size, multifocality, and extrathyroidal extension. Nevertheless, bilaterality and Hashimoto thyroiditis were irrelevant to CLNM. For multivariate analysis, male gender, age <55, and the increase in tumor size resulted as independent predictive factors for CLNM. The results are shown in Table 4. Univariate analysis showed a statistically significant association between LLNM and the increase in tumor size, extrathyroidal extension, and CLNM. In contrast, no significant association was found between LLNM and gender, age, multifocality, and bilaterality. In the multivariate analysis, LLNM was associated with an increase in tumor size and CLNM (Table 5). According to the previous studies, approximately 80% of LLNM was found in patients with ≥2 positive central lymph nodes [13–15]. We tested this hypothesis in the present study, and our results revealed that the number of positive central lymph nodes had a positive correlation with the incidence of LLNM (results are shown in Figures 1 and 2).

## 4. Discussion

The incidence of thyroid cancer has been increasing faster than any other cancer over recent years, especially in developed nations, where people have high access to healthcare [16]. PTC, the most common thyroid malignancy, is a lymphotropic tumor that frequently presents with neck node metastases [17]. More than 99% of PTC patients have a 10-year-survival rate [1]. Nevertheless, some patients suffer from recurrence, reoperation, and disease-specific mortality.

CLNM is common in PTC, with an occurrence rate that ranges from 18% to 90% [4, 5, 14, 18, 19]. We focused on the association of LNM with the clinicopathological characteristics in this study. The male gender, age <55, and the increase in tumor size were closely related to the probability of LNM and CLNM. This finding is consistent with the data reported [20–23]. Those patients with an increase in tumor size and CLNM were extremely prone to LLNM. Interestingly, we found multifocality only was an independent risk factor for LNM, rather than for CLNM and LLNM. This finding is inconsistent with previous studies [23–26]. Therefore, for male and younger patients, we should pay more attention to LNM, especially CLNM. For patients with larger tumor size, we should pay attention not only to CLNM but also to LLNM.

In this study, we found CLNM was an independent risk factor for LLNM, and the rising number of positive central lymph nodes increased the incidence of LLNM. The relationship between positive central lymph nodes and LLNM may be explained by the dissemination of PTC through the lymphatic system [27, 28]. Lymph node metastases tend to occur in consecutive orders from the central compartment, followed by the lateral compartment and then mediastinal lymph nodes [6, 29]. Metastases in the lateral compartment without positive central lymph nodes are very rare [30]. In our study, only 21 patients had skip metastasis. Central lymph node dissection is seen as an effective approach to cut off lymphatic flow to lateral lymph nodes [31]. Some studies have shown that their surgical removal of lymph node metastases improves survival rates [32, 33].

The US is regarded as a routine method to evaluate metastasis in lymph nodes. However, the sensitivity of the US in the evaluation of CLNM is poor, and lymph node metastases of level VI are not detectable preoperatively [34, 35]. In our study, 453 patients (40.9%) without evidence of CLNM following the US examination had cervical lymphatic invasion, which was confirmed by postoperation histopathologic examination. In a total of 144 patients with evidence of LLNM by US examination, 117 patients (81.3%) had lateral lymph node metastases that were also confirmed by histopathologic examination. The huge gaps that were observed between US examination forecasting and CLNM were unexpected.

On the other hand, numerous studies tried to identify what kind of PTC patients were more likely to have CLNM reporting unsatisfactory results and roughly identifying the same risk factors, including male gender, younger age, the increase in tumor size, and so on [10, 13, 31, 36–38]. In the present study, we also evaluated the predictive factors finding no new results. Currently, there are no prominent or particular indexes that could predict CLNM.

For PTC patients, therapeutic CLND is always necessary, while the controversy mainly arises around P-CLND. P-CLND has been considered as an overtreatment [11, 39]. Several international guidelines [40–42] have suggested performing P-CLND only in patients with advanced primary tumors (T3 and T4), or if the information is to be used to plan further steps in therapy. However, the TNM staging system sometimes does not necessarily reflect less aggressive disease [43]. Therefore, there are inconsistent views on P-CLND. According to many clinicians, CLND should be considered even in PTC patients without lymphatic invasion [31, 44]. These poor outcomes mainly occur in patients with LNM, especially CLNM [10]. Numerous studies have concluded that reoperation due to recurrence in cervical nodes is associated with an increase in postoperative complications, such as recurrent laryngeal nerve injury and hypoparathyroidism [9, 45]. Reoperation has also been suggested as a risk factor for disease-specific mortality, which is a more serious problem [46]. Furthermore, a second operation is associated with a requirement for general anesthesia, patient stress, and higher medical healthcare cost [47].

In this study, all patients underwent P-CLND, but the rate of postoperative complications potentially caused by P-CLND was not higher than in other studies. As reported, the rate of persistent hypoparathyroidism ranges from 2.6% to 12% [31, 48–51], whereas in this study, 9 patients (2.8%) undergoing total thyroidectomy were observed having persistent hypoparathyroidism. Meanwhile, the rate of transient hypoparathyroidism in this study (6%) was lower than the reported data (ranging from 6.9% to 9.0%) [31, 47]. Among 34 cases with vocal cord palsy, which implied recurrent laryngeal nerve injury, 22 (2%) recovered within 1 to 6 months while the other 12 (1.1%) patients had a persistent injury; while the rate of transient recurrent laryngeal nerve injury ranges from 1.1% to 6.0%, the rate of persistent recurrent laryngeal nerve injury ranges from 0.2% to 3.6% [31, 47–52]. Several previous studies have reported that the incidence of chyle leakage ranges from 0.5% to 8.3% [53–55], whereas in this study, 5 patients (3.5%) presented with chyle leakage and were cured within 20 days. Most of the patients recovered well. According to our results, routine P-CLND does not increase the risk of complications and can help to get a more accurate stage and to avoid the potential risk of the second operation. As a result, P-CLND during thyroid surgery appears as a reasonable management option.

There are some limitations to this study. First, it is a retrospective, single-institution study. Second, it lacks long-term follow-up results, so the data such as disease recurrence rates, postoperative radioiodine studies, thyroglobulin level fluctuation, and disease-free survival are still unknown. Third, the pathological subtypes were not distinguished in statistics, which might cause bias in the results. We will continue collecting clinicopathological data in the long-term follow-up as a consecutive report.

## 5. Conclusion

CLNM is frequently microscopic and not detectable preoperatively. Our results revealed that the probability of CLNM was closely related to the male gender, age <55, and the increase in tumor size. Those patients with an increase in tumor size and CLNM were extremely prone to LLNM. In addition, LLNM was more likely to happen in those with the more positive central lymph nodes. The rates of postoperative complications potentially caused by CLND were lower than in other studies. Routine P-CLND does not increase the risk of complications and should be considered as a reasonable surgical treatment for PTC.

## Figures and Tables

**Table 1:** Clinicopathologic characteristics of patients.

Characteristics	Number
Number of patients	1107
Age at diagnosis (years)	
<55	816
≥55	291

Gender	
Female	828
Male	279

Tumor size (mm)	
≤5	384
>5, ≤10	366
>10, ≤20	294
>20	63

Multifocal neoplasms	225
Bilateral neoplasms	171
Extrathyroidal extension	120
Hashimoto thyroiditis	195
Lymph node metastasis	474
Central lymph node metastasis	453
Lateral lymph node metastasis	117

Surgery procedure	
Lobectomy plus ipsilateral CLND	789
Total thyroidectomy plus bilateral CLND	174
Total thyroidectomy plus bilateral CLND and unilateral LLND	144

CLND: central lymph node dissection; LLND: lateral lymph node dissection.

**Table 2:** Postoperative complications.

Complications	Number (%)	
	Transient	Persistent
Hypoparathyroidism	67/1107 (6%)	9/318 (2.8%)
Lobectomy	7/789 (0.9%)	0
Total thyroidectomy	60/318 (18.9%)	9/318 (2.8%)
Recurrent laryngeal nerve injury	22/1107 (2%)	12/1107 (1.1%)
Chyle leakage	5/144 (3.5%)	0
Bleeding	2/1107 (0.2%)	0
Hematoma	0	0
Tracheal leak	0	0
Horner's syndrome	0	0

**Table 3:** Univariable and multivariate analysis for LNM.

	Univariate analysis			Multivariate analysis	
Risk factors	LNM+	LNM−	*P* value	*P* value	OR (95% CI)
Age at diagnosis (years)			<0.001	<0.001	2.128 (1.563–2.897)
<55	387	429			
≥55	87	204			
Gender			0.003	<0.001	0.57 (0.421–0.773)
Female	333	495			
Male	141	138			
Tumor size (mm)			<0.001		
≤5	114	270		<0.001	
>5, ≤10	135	231		<0.001	7.252 (3.652–14.399)
>10, ≤20	174	120		<0.001	6.526 (3.301–12.903)
>20	51	12		0.002	3.052 (1.527–6.101)
Multifocal neoplasms			<0.001	0.001	1.712 (1.273–2.370)
Positive	123	102			
Negative	351	531			
Bilateral neoplasms			0.005	0.754	
Positive	90	81			
Negative	384	552			
Extrathyroidal extension			0.001	0.442	
Positive	69	51			
Negative	405	582			
Hashimoto thyroiditis			0.130		
Positive	93	102			
Negative	381	531			

LNM: lymph node metastasis.

**Table 4:** Univariable and multivariate analysis for CLNM.

	Univariate analysis			Multivariate analysis	
Risk factors	CLNM+	CLNM−	*P* value	*P* value	OR (95% CI)
Age at diagnosis (years)			<0.001	<0.001	2.187 (1.606–2.978)
<55	372	444			
≥55	81	210			
Gender			0.012	0.005	0.651 (0.484–0.876)
Female	321	507			
Male	132	147			
Tumor size (mm)			<0.001		
≤5	108	276		<0.001	
>5, ≤10	135	231		<0.001	3.415 (1.896–6.150)
>10, ≤20	168	126		<0.001	2.887 (1.614–5.162)
>20	42	21		0.261	1.403 (0.777–2.533)
Multifocal neoplasms			0.004	0.600	
Positive	111	114			
Negative	342	540			
Bilateral neoplasms			0.062		
Positive	81	90			
Negative	372	564			
Extrathyroidal extension			<0.001	0.617	
Positive	66	54			
Negative	387	600			
Hashimoto thyroiditis			0.247		
Positive	87	108			
Negative	366	546			

CLNM: central lymph node metastasis.

**Table 5:** Univariable and multivariate analysis for LLNM.

	Univariate analysis			Multivariate analysis	
Risk factors	LLNM+	LLNM−	*P* value	*P* value	OR (95% CI)
Age at diagnosis (years)			0.134		
<55	93	723			
≥55	24	267			
Gender			0.429		
Female	84	744			
Male	33	246			
Tumor size (mm)			<0.001		
≤5	15	369		<0.001	
>5, ≤10	21	345		<0.002	13.165 (6.177–28.060)
>10, ≤20	51	243		<0.003	11.946 (5.909–24.151)
>20	30	33		<0.004	4.917 (2.614–9.250)
Multifocal neoplasms			0.958		
Positive	24	201			
Negative	93	789			
Bilateral neoplasms			0.109		
Positive	24	147			
Negative	93	843			
Extrathyroidal extension			0.006	0.445	
Positive	21	96			
Negative	96	894			
Hashimoto thyroiditis			0.503		
Positive	18	177			
Negative	99	813			
CLNM			<0.001	<0.001	5.463 (3.264–9.143)
Positive	96	357			
Negative	21	633			

CLNM: central lymph node metastasis; LLNM: lateral lymph node metastasis.

## Data Availability

The raw clinical data used to support the findings of this study are available from the corresponding author upon request.
